# Transcriptome profiling of *Lymnaea stagnalis* (Gastropoda) for ecoimmunological research

**DOI:** 10.1186/s12864-021-07428-1

**Published:** 2021-03-01

**Authors:** Otto Seppälä, Jean-Claude Walser, Teo Cereghetti, Katri Seppälä, Tiina Salo, Coen M. Adema

**Affiliations:** 1 Institute of Integrative Biology (IBZ), ETH Zürich, 8092 Zürich, Switzerland; 2 Eawag, Swiss Federal Institute of Aquatic Science and Technology, 8600 Dübendorf, Switzerland; 3 Research Department for Limnology, University of Innsbruck, A-5310 Mondsee, Austria; 4 Genetic Diversity Centre (GDC), ETH Zürich, 8092 Zürich, Switzerland; 5 Environmental and Marine Biology, Åbo Akademi University, FI-20520 Turku, Finland; 6 Center for Evolutionary and Theoretical Immunology, Department of Biology, The University of New Mexico, Albuquerque, NM 87131 USA

**Keywords:** Ecological immunology, Great pond snail, Mollusc

## Abstract

**Background:**

Host immune function can contribute to numerous ecological/evolutionary processes. Ecoimmunological studies, however, typically use one/few phenotypic immune assays and thus do not consider the complexity of the immune system. Therefore, “omics” resources that allow quantifying immune activity across multiple pathways are needed for ecoimmunological models. We applied short-read based RNAseq (Illumina NextSeq 500, PE-81) to characterise transcriptome profiles of *Lymnaea stagnalis* (Gastropoda), a multipurpose model snail species. We used a genetically diverse snail stock and exposed individuals to immune elicitors (injury, bacterial/trematode pathogens) and changes in environmental conditions that can alter immune activity (temperature, food availability).

**Results:**

Immune defence factors identified in the de novo assembly covered elements broadly described in other gastropods. For instance, pathogen-recognition receptors (PRR) and lectins activate Toll-like receptor (TLR) pathway and cytokines that regulate cellular and humoral defences. Surprisingly, only modest diversity of antimicrobial peptides and fibrinogen related proteins were detected when compared with other taxa. Additionally, multiple defence factors that may contribute to the phenotypic immune assays used to quantify antibacterial activity and phenoloxidase (PO)/melanisation-type reaction in this species were found. Experimental treatments revealed factors from non-self recognition (lectins) and signalling (TLR pathway, cytokines) to effectors (e.g., antibacterial proteins, PO enzymes) whose transcription depended on immune stimuli and environmental conditions, as well as components of snail physiology/metabolism that may drive these effects. Interestingly, the transcription of many factors (e.g., PRR, lectins, cytokines, PO enzymes, antibacterial proteins) showed high among-individual variation.

**Conclusions:**

Our results indicate several uniform aspects of gastropod immunity, but also apparent differences between *L. stagnalis* and some previously examined taxa. Interestingly, in addition to immune defence factors that responded to immune elicitors and changes in environmental conditions, many factors showed high among-individual variation across experimental snails. We propose that such factors are highly important to be included in future ecoimmunological studies because they may be the key determinants of differences in parasite resistance among individuals both within and between natural snail populations.

**Supplementary Information:**

The online version contains supplementary material available at 10.1186/s12864-021-07428-1.

## Background

Several fields of ecology and evolution increasingly recognise host immune function as an essential contributor to biological processes (see [1]). For instance, the immune system plays critical roles in life-history evolution [2, 3], sexual selection [4, 5], and responses/adaptations of organisms to environmental change [6–8]. This recognition has given rise to an interdisciplinary field of ecological immunology (or ecoimmunology; see [9]). Ecoimmunological studies, especially in invertebrates, typically measure the end products of one or few immunological cascades that are controlled by several genes (e.g., [10, 11]). Thus, the field relies on quantitative genetic theory, initially motivated by the assumed simplicity of innate-type invertebrate immune systems with non-specific recognition and killing mechanisms. Over the recent decades, however, comparative immunology with aid from genomics, has shown invertebrate immune systems to be complex and diversified across the phyla (e.g., [12–16]). In fruit flies, for instance, specific immune pathways respond towards Gram-positive and Gram-negative bacteria, as well as fungi [17–19].

The realised complexities of invertebrate immune systems provide new challenges and opportunities for studies focusing on ecological and evolutionary questions on immune function. This is because typical ecoimmunological studies that focus on one/few immunological mechanisms are incapable of describing the multivariate “immune phenotypes” (sensu [20]) of organisms. That, however, would be important because each immunological pathway can respond differently to various selective agents (e.g., parasite/pathogen species) and may be traded-off with different physiological, life-history, and other immune traits [10, 14, 21–23]. Additionally, the expression of immune traits, as well as the associated trade-offs, may depend on environmental conditions such as resource availability and temperature (e.g., [8, 24, 25]), which could be due to stress-related responses or altered metabolism. Therefore, a comprehensive characterisation of different immunological and physiological mechanisms, as feasible by genomics-type approaches, in ecologically relevant experiments utilising invertebrates is essential. This expansion has been done successfully in some insects such as bumblebee (e.g., [26, 27]) and red flour beetle [28, 29]. Considering the extensive diversity of invertebrate phyla, development and utilisation of such genomic data also in other taxonomic groups would further expand the potential of ecoimmunology.

Mollusca is the second-largest animal phylum after Arthropoda. In this phylum, Gastropoda represents the largest taxonomic class with 40,000 to 150,000 living species [30]. Gastropods inhabit aquatic and terrestrial habitats, and incur disease from viruses and bacteria (e.g., [31–33]) but also from specialist flatworm parasites called digenetic trematodes [34, 35]. Trematodes have complex life cycles that typically involve a gastropod as an intermediate host. In snails, trematodes reproduce asexually to produce transmission stages that infect the next host in the life cycle (another intermediate host or a definitive host). Most of the attention on molecular immunology in gastropods has focused on *Biomphalaria glabrata* (Planorbidae, Hygrophila, Panpulmonata), a tropical freshwater snail that transmits the human blood fluke, *Schistosoma mansoni* [36–38]. Attention to other species has been comparatively limited although several gastropods, including pond snails of the family Lymnaeidae (Hygrophila, Panpulmonata), transmit medically and veterinary-relevant parasites (e.g., *Fasciola hepatica*, *Diplostomum* spp., *Trichobilharzia* spp., [39–41]) in temperate regions. In this family, diversity and function of circulating defence cells, haemocytes, have been investigated in the great pond snail, *Lymnaea stagnalis* [42–49]. Additionally, a draft genome of *L. stagnalis* is available [50].

Here, we applied short-read based RNAseq to characterise transcriptome profiles of *L. stagnalis* exposed to various immune elicitors (injury, bacterial and trematode pathogens) and changes in environmental conditions (temperature, food availability) using a genetically diverse laboratory population of snails. *Lymnaea stagnalis* is a model organism in multiple biological disciplines (reviewed in [51]), including ecological immunology (e.g., [8, 52–56]). Earlier ecoimmunological work, however, employs only a few phenotypic immune assays. Therefore, our data analyses focused on examining the suitability of a broad range of candidate immune genes for quantifying snail immune activity. Additionally, we investigated components of snail physiology/metabolism that may be related to immune responses. In a two-step process, we first annotated the transcriptome using previously characterised immune/physiology-related proteins/genes from other organisms. After that, we evaluated variation in the transcription of those candidates to detect transcripts that responded to experimental treatments and/or that showed high among-individual variation. We propose that the use of candidate genes exhibiting high variation in transcription allows a comprehensive examination of variation in polymorphic snail immune responses in future ecoimmunological studies.

## Results

### Sequencing and assembly

Sequencing (Illumina NextSeq 500 platform) of all 48 libraries (generated from RNA samples extracted from homogenised whole-body tissues) yielded a total of 1.08 billion paired-end 81 nt long reads (PE-81). The raw reads representing a total of 175.2 Gb sequence data were deposited in the NCBI Sequence Read Archive (accession number PRJNA664475). The de novo assembly (trinity v2.0.6) of the reads from nine libraries (one per each experimental treatment; treatments: untreated, anaesthesia, wounding, injection with *Escherichia coli*, injection with *Micrococcus lysodeikticus*, injection with healthy snail tissue, injection with trematode-infected snail tissue, elevated temperature, food deprivation) yielded 264,746 transcripts with N50 of 1589 nt, mean length of 551 nt, and median length of 188 nt (146.0 Mb in total). These transcripts included 68,473 open reading frames (ORFs) that were longer than 100 aa (TransDecoder v3.0.1). From this initial full assembly, the removal of likely contaminant sequences that lacked a significant similarity hit with *L. stagnalis* genome (GenBank accession number GCA_900036025.1) and that were not previously characterised from this species, yielded a final reference transcriptome (139.1 Mb) consisting of 226,116 contigs with a mean transcript length of 615 nt (assembly available in 10.5281/zenodo.4044169). BUSCO analysis indicated high completeness of the reference transcriptome based on the detection of 98.8% (complete plus partial) of the set of metazoan universal single-copy orthologs [complete: 96.3% (duplicated: 28.2%), fragmented: 2.5%, missing: 1.2%, *N* = 978].

### Sequences supporting species identification

The reference transcriptome included sequences that represent parts of the snail rDNA cassette, including the complete ITS1 and ITS2 regions (GenBank accession numbers MT864603 and MT864602, respectively). *Lymnaea stagnalis* was confirmed as the species identity of the experimental snails by the highest sequence similarities (BLASTN) of these sequences with GenBank entries from this species. Similarly, snails exposed to tissue extracts from trematode-infected gonads provided LSU-derived sequences with the highest similarity with Clinostomidae and Plagiorchiidae families of trematodes (GenBank accession numbers MT872505 and MT872506, respectively), which confirms the morphological identification of cercaria released by the donor snails.

### Transcriptome annotation

**Fig. 1:**
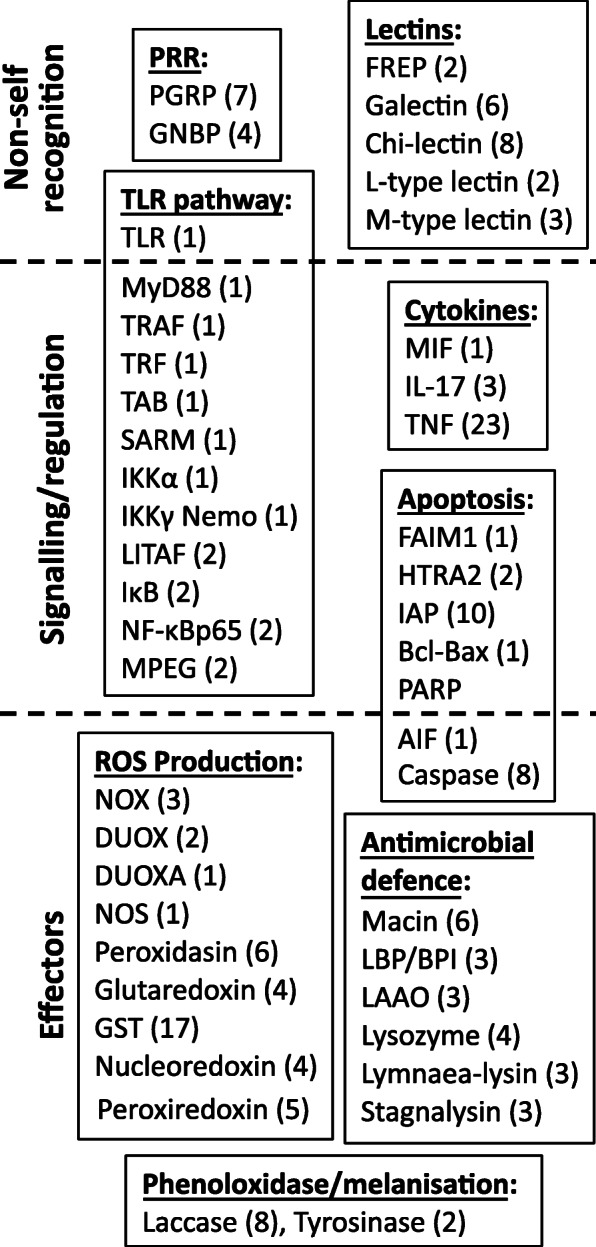
Summary of the identified immune defence factors in *Lymnaea stagnalis* reference transcriptome. Factors are organised across different immunological mechanisms/pathways and steps of the immune response (i.e., non-self recognition, signalling/regulation, effectors). Numbers in brackets show how many transcripts with unique open reading frames (ORFs) were detected from those factors for which determining the completeness of ORFs was possible

The mining of the *L. stagnalis* reference transcriptome (226,116 contigs) yielded numerous factors that contribute to immune responses from non-self recognition to the elimination of pathogens (Fig. 1, Additional file 1). For accommodation of PRRs, *L. stagnalis* expressed seven variants (i.e., transcripts with unique ORFs) of long-form (no short forms) peptidoglycan recognition proteins (PGRPs), four variants of Gram-negative bacteria binding-proteins (GNBPs) and a repertoire of lectins. Representatives of the detected lectin families included two FREPs of the VIgLs (variable immunoglobulin and lectin-domain-containing molecules), galectins comprising of either one, two or four carbohydrate recognition domains, multiple Chi-lectins, as well as L- and M-type lectins. The latter two families may, however, function mostly in housekeeping roles.

Along with one TLR sequence encoding the canonical architecture of tandemly arranged leucine-rich repeats (LRRs), a transmembrane region and a C-terminal Toll-interleukin receptor (TIR) domain, the associated signalling NFκB pathway for immune activation was represented by multiple components such as adaptor proteins for intracellular signalling downstream of the receptor (MyD88, TRAF), transcription factor (Rel protein) NF-κB, and various regulators (e.g., SARM, IκB). Additionally, three categories of cytokines for intercellular communication (including immune responses and inflammation) were detected; one variant of macrophage migration inhibitory factor (MIF), three variants of interleukin 17 (IL-17) and 23 variants of tumor necrosis factor (TNF).

The search of the complete reference transcriptome for antimicrobial defences yielded a single family of macin antimicrobial peptides (AMPs, 6 variants), as well as several families of antimicrobial proteins, with an abundant representation of lipopolysaccharide-binding /bactericidal permeability-increasing proteins (LBP/BPI, 3 variants), L-amino acid oxidases (LAAOs, 3 variants), lysozymes (4 variants), and transcripts encoding for cytolytic β pore-forming toxins. These latter sequences were designated Lymnaea-lysins (3 variants) and stagnalysins (3 variants), in analogy to the orthologous Biomphalysins and glabralysins described from *B. glabrata* [57, 58]. Central components from a gene network facilitating the production and management of reactive oxygen species for oxidative killing mechanisms were detected, including the membrane-bound enzyme complex NADPH-oxidase (NOX, with Cytochrome B 245 as the main component, 3 variants) that produces superoxide anions, as well as dual oxidases (DUOX, 2 variants) that catalyse the synthesis of anion superoxide and hydrogen peroxide. Moreover, dual oxidase maturation factor (DUOXA, 1 variant) that activates DUOX was found, as well as additional proteins involved in the biosynthesis of other oxidative compounds. Furthermore, eight variants of laccase and two of tyrosinase that can contribute to PO/melanisation-type defence were recorded. The search also yielded transcripts encoding for antioxidant enzymes and proteins regulating oxidative damage. These transcripts included two types of superoxidase dismutase enzymes against ROS [SOD (2 variants) and MnSOD (1 variant)], catalase (CAT, 2 variants), glutathione peroxidase (3 variant), and glutathione reductase (1 variant).

Our analysis also revealed transcripts that encode for proteins involved in apoptosis (programmed cell death), a process that also functions in internal defence responses. Identified transcripts included FAIM1 that regulates the extrinsic signalling pathway leading to apoptosis. Representatives of the intrinsic signalling pathway included serine protease HTRA2 (2 variants) that contribute to the loss of the mitochondrial transmembrane potential, one apoptosis-inducing factor (AIF) that causes DNA fragmentation and chromatin condensation, and factors that regulate these processes (Baculovirus IAP Repeat domain-containing caspase inhibitors, Bcl-Bax, PARP). Finally, eight variants of caspases that destroy critical cellular proteins were detected.

The search for response factors related to environmental change yielded two families of heat shock proteins that are induced by a variety of stressors: HSP70 (5 variants) and HSP90 (1 variant). Along with the HSPs, a heat shock factor (HSF, 1 variant) that regulates the expression of HSPs was identified. Additionally, our analysis yielded transcripts linked to invertebrate metabolism. These included one protein phosphatase (PP1) that controls for cellular processes linked to metabolism, gene transcription and translation, cell movement and apoptosis, and eight variants of ubiquitin, a regulatory protein that marks proteins, for instance, for degradation and recycling by proteasome and affects protein activity. Furthermore, transcripts encoding for ferritin (3 variants) that participates in iron transport and storage in invertebrates and protects organisms from iron-induced oxidative stress, and alcohol dehydrogenase (ADH, 1 variant) that catalyses the interconversion between alcohols and aldehydes or ketones were identified. Lastly, the search yielded transcripts linked to the estrogen-related receptor (ERR, 2 variants) and retinoid acid receptor (RXR, 3 variants) that regulate, for instance, oxidative metabolism, energy homeostasis, and imposex development in invertebrates.

### Transcriptomic responses to experimental treatments

**Fig. 2:**
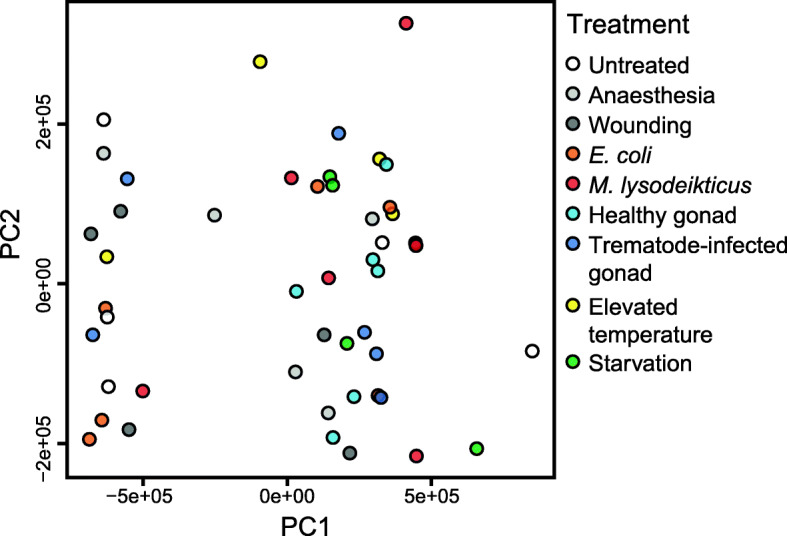
Principal component analysis (PCA) plot showing variation in transcriptome-wide expression profiles of the experimental snails. The first two principal components (PCs) after internal normalisation in Sleuth are used. PCA plots for the first five PCs, as well as the proportion of total variance each of them explained in the data, are presented in Additional file 2

**Fig. 3:**
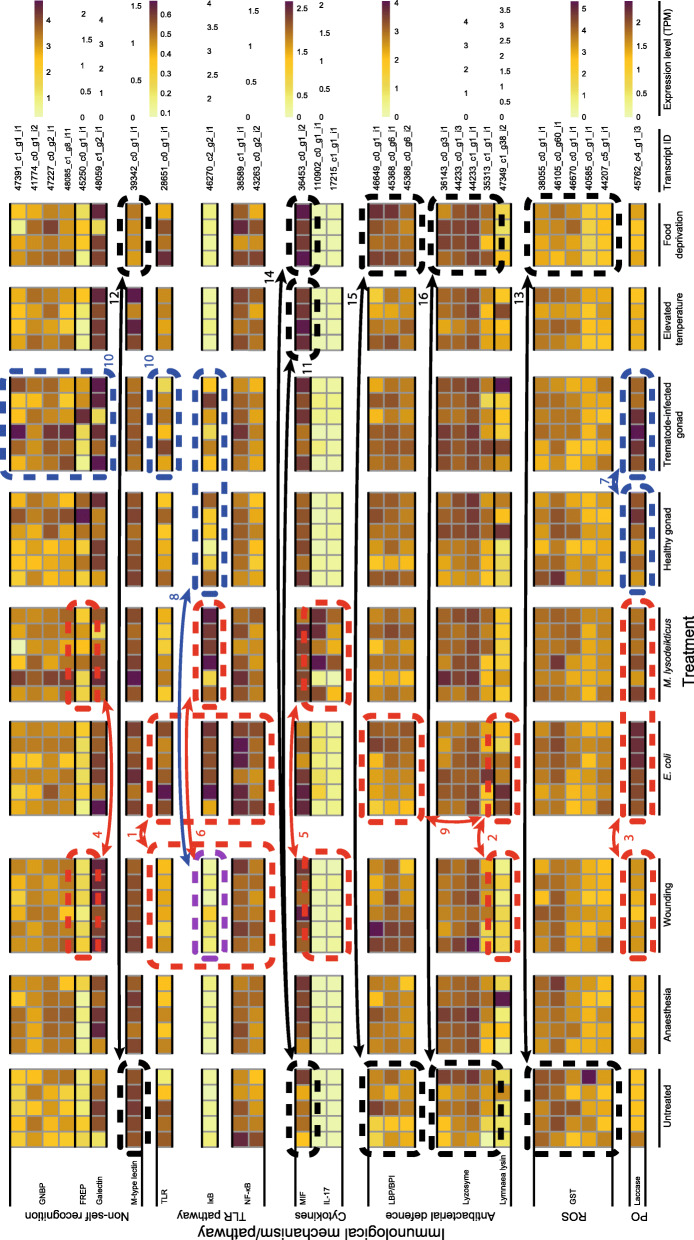
Expression levels of selected transcripts that represent different components of the immune system. Heatmap shows transcripts that deviated in their transcription between certain experimental treatments and their specific controls and components of the immune system in which individuals expressed distinct transcripts. Examined immunological pathways/mechanisms included non-self recognition, Toll-like receptor (TLR) signaling pathway, cytokines, antibacterial defence, production of reactive oxygen species (ROS), and phenoloxidase (PO)/melanisation-type reaction. Transcripts within each pathway/mechanism are clustered according to their similarity. Heatmap shows the variation for each factor among all experimental snails (each column represents one snail) using its dynamic range in units of transcripts per million (TPM). Red (injections with bacteria), blue (injections with snail/trematode tissue extracts), purple (injections with bacteria and snail/trematode tissue extracts) and black (exposures to environmental change) rectangles (dashed line), arrows and numbers refer to the specific results mentioned in the text

Plots of principal component (PC) scores for the transcriptome-wide expression profiles of individual snails did not reveal any grouping based on experimental treatments or outlier libraries that deviated from others (Fig. 2, Additional file 2). Heatmaps for the transcription of the annotated immune factors (see Additional file 1) across experimental treatments indicated some immune-elicitor specific responses (Fig. 3). For instance, injection with lyophilised *E. coli* cells increased the transcription of TLR, as well as of some components of the TLR signalling pathway (IκB, NF-κB), when compared to the levels in snails injected with physiological saline (comparison 1 in Fig. 3, Additional file 3). Additionally, exposure to *E. coli* increased the transcription of effectors representing antibacterial defence [Lymnaea-lysins (4 out of 6 individuals); comparison 2 in Fig. 3, Additional file 4] and PO/melanisation-type reaction (laccase; comparison 3 in Fig. 3, Additional file 5). Exposure of snails to lyophilised *M. lysodeikticus* cells activated the transcription of the lectin FREP (3 out of 6 individuals; comparison 4 in Fig. 3, Additional file 6), the cytokine IL-17 (comparison 5 in Fig. 3, Additional file 7), one component of the TLR signalling pathway (IκB; comparison 6 in Fig. 3, Additional file 3), and the effector laccase (comparison 3 in Fig. 3, Additional file 5). The injection of soluble extracts from trematode-infected snail tissue increased the transcription of laccase (comparison 7 in Fig. 3, Additional file 5) when compared to the snails challenged with extracts from healthy snail tissue. Also, IκB was activated in some individuals exposed to extracts of both healthy and trematode-infected snail tissue (comparison 8 in Fig. 3, Additional file 3). Additionally, wounding led to upregulation in the transcription of DUOXA (Additional file 8) that contributes to ROS production. These responses conform to the notions of their importance for invertebrate immune function.

Befitting the innate type immunity of invertebrates, the transcription of other immune factors did not show systematic differences among immune activation treatments (Additional files 3-9). However, the transcription of many factors showed high among-individual variation within treatment groups. This was the case for PRRs GNBP (Additional file 6), galectin (Additional file 6) and TLR (Additional file 3), as well as for some components of the TLR signalling pathway (Myd88, TRAF, NF-κB p65; Additional file 3), cytokines MIF and TNF (Additional file 7), several components of ROS production (NOX, DUOXA, GST, nucleoredoxin, NOS, peroxidasin; Additional file 8), the effector tyrosinase (PO/melanisation-type reaction; Additional file 5), factors regulating apoptosis (HTRA2, AIF, Bcl-Bax, PARP; Additional file 9), and all examined antibacterial defence factors (Additional file 4). A few immune factors showed high among-individual variation that was limited to certain immune activation treatments: lectin FREP (Additional file 6), IκB (TLR pathway; Additional file 3), DUOX (ROS production; Additional file 8), laccase (PO/melanisation-type reaction; Additional file 5) and the cytokine IL-17 (Additional file 7). Interestingly, within certain components of the immune system, individual snails expressed distinct gene transcripts, which suggests different defence strategies against pathogens. For instance, snails challenged with *E. coli* showed high transcription in two to four of the examined six antibacterial defence factors and the factors with high levels were different among individuals (Additional file 4). Most strikingly, individuals with the highest transcription of Lymnaea lysins showed the lowest transcription of LBP/BPI and vice versa (comparison 9 in Fig. 3). Similarly, snails exposed to trematode-infected snail tissue extracts showed high transcription in only one to two of the examined non-self recognition components (GNBP, FREP, galectins, TLR), and the expressed factors were different among individuals (areas 10 in Fig. 3).

**Fig. 4:**
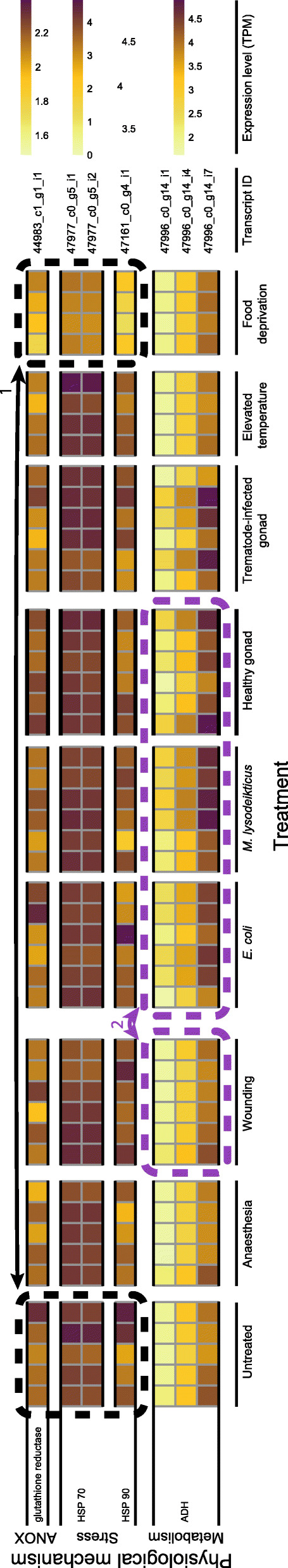
Expression levels of selected transcripts that represent antioxidation (ANOX), stress responses and metabolism. Heatmap shows transcripts that deviated in their transcription between certain experimental treatments and their specific controls. Transcripts within each pathway/mechanism are clustered according to their similarity. Heatmap shows the variation for each factor among all experimental snails (each column represents one snail) using the dynamic range in units of transcripts per million (TPM). Purple (immune challenge) and black (exposures to environmental change) rectangles (dashed line), arrows and numbers refer to the specific results mentioned in the text

The examined environmental changes apart from immune challenge affected several components of the snail immune system. The elevated temperature increased the transcription of the cytokine MIF (comparison 11 in Fig. 3, Additional file 7) when compared to untreated snails. Food deprivation, on the other hand, downregulated the transcription of an M-type lectin (comparison 12 in Fig. 3, Additional file 6), and glutathione S-transferase (GST, comparison 13 in Fig. 3, Additional file 8), but enhanced the transcription of MIF (comparison 14 in Fig. 3, Additional file 7), LBP/BPI (comparison 15 in Fig. 3, Additional file 4) and lysozyme (comparison 16 in Fig. 3, Additional file 4). Additionally, some of the factors that contribute to stress-responses, antioxidation and metabolism were found to be affected by the experimental treatments (Fig. 4). First, heat shock proteins (both HSP70 and HSP90; comparison 1 in Fig. 4, Additional file 10) and glutathione reductase (comparison 1 in Fig. 4, Additional file 11) showed reduced transcription under food deprivation. Exposure of snails to immune elicitors (injection with lyophilised *E. coli* and *M. lysodeikticus* cells, injection with healthy snail gonad) increased the transcription of ADH (comparison 2 in Fig. 4, Additional file 12) when compared to the snails injected with physiological saline. Similarly to the immune defence genes, high among-individual variation was seen in some annotated factors with a potential role as antioxidant enzymes [SOD (all treatments), MnSOD (some treatments), glutathione reductase (most immune activation treatments); Additional file 11] or in snail metabolism [PP1 (most treatments), ADH (most immune activations), RXR, (most treatments); Additional file 12].

## Discussion

This study aimed to produce a resource for future ecoimmunological research on the freshwater snail *L. stagnalis* by recording snail transcriptomic responses to immune challenges and environmental changes, with a focus on know immune factors. Earlier work on snail ecoimmunology has relied on quantifying a narrow subset of phenotypic immune defence traits. Those traits, however, respond differently to immune elicitors [59] and environmental conditions (e.g., [8, 52, 55, 60]), and they contribute differently to snail fitness [54]. Additionally, contrary to the earlier expectation of simple innate-immune system in invertebrates (i.e., non-specific defence mechanisms) the recent development in comparative immunology and genomics/transcriptomics has revealed invertebrate immune systems to be highly complex, diverse, and also specific against different parasite types (e.g., [12–16]). Together these findings call for ecological experiments that quantify snail “immune phenotypes” across a wide range of immunological mechanisms. Only such studies may evaluate the role of snail immune function as a whole in ecological and evolutionary processes.

In this study, the samples for transcriptome sequencing were extracted from whole-body tissues of individual snails to avoid any bias in the recovery of immune and stress-response factors that are expressed in specific tissues or cell types, even if this may lead to reduced sensitivity to detect rare transcripts (see e.g., [38, 61]). Focusing on specific tissues/organs could be more sensitive compared to the whole-body approach when comparing experimental treatments, but we chose to use a measure that describes an individual’s overall immune activity. The reference transcriptome assembled from nine individuals exposed to different experimental treatments may not fully represent the genome content of *L. stagnalis* because mRNA-based approaches only capture genes that are actively expressed. BUSCO analysis, however, indicated representation of 98.8% of the universally shared metazoan genes in the assembly. Therefore, the reference transcriptome can be assumed to be highly comprehensive. Additionally, the use of a genetically diverse lab stock may have led to the capture of allelic variants of genes that do not all occur within single snail individuals*,* yet exist in natural populations where they may contribute differently to organismal fitness. This is supported by the high proportion (28.2%) of core BUSCO genes that were found with more than one copy.

Along with the aim to support future ecoimmunological research, the characterisation of immune genes of *L. stagnalis* broadens comparative immunology of aquatic pulmonate snails (Hygrophila), previously available only for a few species from the families Lymnaeaidae, Physideae and Planorbidae [38, 62, 63]. The organisation of antimicrobial defences in *L. stagnalis* is in line with hygrophilid snails that all show considerable diversity of antimicrobial proteins (LBP/BPI, LAAO, lysozyme, lymnaealysin, cytolytic β pore-forming toxins), contrasted by a modest number of AMP genes (a single family of 5 macin-type transcripts in *L. stagnalis*). This is remarkable because of the great diversity and numbers of AMP genes and gene families that are usually recorded from other organisms, including bivalve molluscs (e.g., [64]). Perhaps hygrophylid gastropods employ novel categories of AMPs that remain to be characterised, but it appears that these snails have a unique approach to dealing with invading microbes.

The recovery of *L. stagnalis* FREPs is consistent with the distribution of VIgLs as likely multimeric immune recognition factors across Gastropoda [65]. The low number of FREPs in *L. stagnalis* is similar to that of the physid *Physella acuta* (Schultz et al., 2017) but differs from the planorbid *B. glabrata* that generates unique repertoires of multiple FREPs at the individual level [66]. Furthermore, the transcriptomic responses of *L. stagnalis* to the introduction of extracts of healthy gonad tissue from other individuals (used as a control for injection with trematode-infected gonad extracts) was remarkably similar to those elicited by wounding alone. The lack of an immune response to allotypic tissue extracts suggests the absence of (acute) allograft rejection in *L. stagnalis*, just as it lacks from closely related planorbid snails [67, 68]. Together these findings suggest that snail immune function has evolved in a lineage-specific manner. However, our study supports the view that the main aspects of innate immunity are conserved throughout animal evolution [69]. For example, lectins and GNBP receptors are available to detect pathogens and signal through the TLR pathway and cytokines to activate defence responses that encompass cellular (e.g., ROS production) and humoral (antimicrobial proteins) branches of the immune system.

The obtained detailed information regarding the molecular immunology of *L. stagnalis* can now be integrated into future ecoimmunological investigations. Studies on *L. stagnalis* typically measure two phenotypic immune defence traits from snail haemolymph, namely antibacterial activity and PO-like activity (e.g., [8, 52–56, 60]). Antibacterial activity is quantified as a reduction in optical density of a solution in which lyophilised *E. coli* cells are mixed with snail haemolymph (see [52, 59]). The current study identified several different effector proteins that may contribute to antibacterial responses. These included AMPs, LBP/BPI, LAAOs, lysozymes and cytolytic β pore-forming toxins. Therefore, the phenotypic antibacterial activity assay is likely to estimate the combined activity of several components. The other commonly studied phenotypic immune defence trait, PO-like activity, measures an increase in optical density of a solution in which PO enzymes from snail haemolymph oxidise the substrate L-dopa (see [52, 59]). This assay may also measure the combined activity of different factors (see [70]). Our study revealed two categories of PO enzymes, laccase and tyrosinase, suggesting that they may be the most important contributors to the melanisation-type reaction in *L. stagnalis*. Further tests at the phenotypic level using enzyme-specific substrates can estimate their relative importance in snail immune function (see [70]).

Visual examination of the transcription patterns of the annotated immune defence factors suggested different responses both against Gram-negative (*E. coli*) and Gram-positive (*M. lysodeikticus*) bacteria. Exposure to *E. coli* activated TLR pathway and the transcription of two effectors, Lymnaea-lysins (antibacterial activity) and laccase (PO activity). Patterns after exposure to *M. lysodeikticus* suggested upregulation in one FREP lectin and cytokine IL-17. The heat map analysis also indicated trematode-infected snail tissue extracts to activate the transcription of laccase. The apparent pathogen-specificity of some immune components is in line with previous findings in other invertebrates (e.g., [14, 17–19, 21]). Two of the annotated factors (laccase, IκB), however, showed broader responses to various immune elicitors. IκB (regulator of TLR signalling), showed increased transcription to all immune challenges except wounding, although this effect was not seen in all individuals. This finding suggests that the TLR pathway generally controls immune responses in *L. stagnalis*, similar as in other organisms [71, 72].

Based on the heat maps, manipulating the environmental conditions altered the transcription of some immune factors. Elevated temperature (25 °C vs. 20 °C) activated cytokine MIF and antibacterial lysozymes. This result differs from previous observations of high temperature reducing antibacterial and PO-like activity of snail haemolymph at the phenotypic level (e.g., [8, 53, 55]). In those studies, however, adverse effects were observed after a one-week exposure to high temperature [60]. In this study, the exposure lasted for two days. Together these findings suggest that only prolonged exposure to high temperature negatively impacts snail immune function. Instead, short-term exposure to high temperature could have positive effects, for example, by increasing general performance through faster metabolic rate. In fact, high temperature initially increases snail growth rate and reproductive output [60]. Food deprivation had both positive (MIF, LBP/BPI, lysozymes) and negative (M-type lectins, glutathione) effects on the transcription of immune factors. Previously, food deprivation has been reported to reduce snail immune activity at the phenotypic level, although also this effect depends on the duration of reduced food availability [52].

The transcription patterns did not indicate activation of the heat shock proteins at elevated temperature (25 °C vs. 20 °C). This finding suggests that the high-temperature treatment may not have been highly stressful to snails. Although 25 °C is above the thermal optimum of adult *L. stagnalis* snails (21–23 °C depending on the trait), it was still not close to the critically high temperature of this species [56]. Additionally, the exposure time was short compared to earlier studies (see the previous paragraph) and to a typical 8.4-day summer heatwave the snails are exposed to in nature [73]. Interestingly, transcription of the heat shock proteins was reduced under food deprivation, which indicates their general role in stress responses and that the resource level of snails could affect their ability to tolerate exposure to high temperature. Such interactive effects should be examined in future studies. Furthermore, exposure of snails to both Gram-negative and Gram-positive bacteria increased the transcription of ADH. This suggests that immune challenge can alter the metabolic rate of snails. However, although immune activation is often expected to increase metabolic activity (reviewed in [74]), other annotated factors with a potential role in metabolism were not affected by immune challenge treatments.

Our study was conducted using snails that originated from a genetically diverse laboratory stock that was formed by initially combining individuals from different natural populations [54]. Genetically variable snails were used to identify genes that show high among-individual variation in transcription. Such variation may arise, for example, from differences in the genetic background (expressed within and/or among populations) and physiological condition of snails. Identifying genes that vary in their transcription among individuals is very important for ecological and evolutionary studies that focus on understanding the sources and consequences of trait variation. Understanding among-individual variation can be critical because trait evolution may depend more strongly on variation in gene expression than differences in protein-coding sequences [75, 76]. Moreover, the suitability of considering transcription as a “trait”, and that it evolves in natural populations has been demonstrated in yeast (e.g., [77, 78]), fruit fly (e.g., [79, 80]), and fish (e.g., [81–83]). Among-individual variation in transcription is, however, easily overlooked in experiments that use genetically as homogeneous individuals as possible (e.g., one clone/inbred strain).

The heat map analysis suggested high variation in transcription among snail individuals in several components of the snail immune system from pathogen recognition (GNBP, lectins, TLR), to signalling/regulation (TLR pathway, cytokines, ROS production, apoptosis) and effectors (laccase, tyrosinase, antibacterial proteins). In several cases, such variation was seen even among untreated snails (e.g., PGRP, TLR, Myd88, MIF, LBP/BPI, LAAOs, lysozymes, NOX, DUOX, TNF receptor). Two potential explanations are proposed for the latter finding. First, control snails may have harboured obscure infections from opportunistic microbes or viruses that activate snail immune function [59], leading to variation in transcription profiles. Second, these components of the snail immune system are expressed at a constant level within each individual, but the actual level varies among individuals [84], perhaps due to differences among alleles that they carry. This would fit with innate type immunity, the expression levels reflecting the immediate capacity to respond to invading pathogens. Nevertheless, all the immune factors that showed high among-individual variation in transcription are potentially interesting targets for future ecoimmunological research. To our knowledge, such variation has been utilised in ecoimmunology only in bumblebee [26, 27, 85, 86]. In that species, condition dependence of immune defence and genetic specificity determining the outcome of a host-parasite interaction have been examined at the transcription level. Similar variation may underlie the observation that parasite exposure does not yield infection in all snails of a generally susceptible strain in the *B. glabrata-S. mansoni* interaction [87].

Despite high among-individual variation in the transcription of many components of the snail immune system, our data did not reveal any individuals with generally very low or very high immune activity across the annotated factors. However, individuals within some of the immune activation treatments showed investment in different components of defence. For example, each snail exposed to *E. coli* showed high transcription in two to four of the examined six antibacterial defence factors. Interestingly, which of these six factors showed high expression levels varied among individuals. Similarly, in snails exposed to trematode-infected snail tissue extracts, each individual typically showed high transcription in only one, but not the same of the examined non-self recognition components (GNBP, FREP, galectins, TLR). Yet other immune properties vary among individuals both within field- and laboratory-maintained *L. stagnalis* populations. Van der Knaap et al. [88, 89] described two genetically-defined types of *L. stagnalis*, with “type I” snails having a particular type of lectin-mediated immune recognition that was absent in the majority of snails, designated as type II. These observations suggest that snails may show different immune defence strategies that lead to diverse responses even under a similar immune challenge. Nevertheless, the data presented in this article does not allow estimating whether the observed among-individual variation in transcription arises from differences expressed within and/or among populations. Both sources of variation are important to consider, which calls for studies investigating variation in the transcription of different immunological mechanisms and their contribution to snail performance (e.g., survival, fecundity) at both within- and among-population scales. Such studies would, for example, allow examining evolution as a within-population process (e.g., natural selection) and an outcome such as differentiation in immune activity among populations that have experienced different pathogen histories.

## Conclusions

Our results confirm uniform aspects of gastropod/molluscan immunity, but also demonstrate apparent differences between *L. stagnalis* and some previously examined taxa. For instance, PRRs and lectins are present to detect pathogens and signal through the TLR pathway and cytokines to activate both cellular and humoral defence mechanisms. However, contrary to the high number of AMPs in other organisms, including molluscs, only a modest number of macin type AMPs was identified in *L. stagnalis*. Similarly, the low number of FREPs is comparable to *P. acuta* but differs from *B. glabrata* that shows high FREP diversity. Our data also indicate that various defence factors can contribute to the phenotypic immunological assays (antibacterial activity and PO-like activity of haemolymph) commonly used in earlier ecoimmunological research. Additionally, immune activation treatments (bacteria, trematodes) and manipulations of environmental conditions (ambient temperature, resource availability) revealed factors that contribute to snail immune responses (TLR pathway, FREP, cytokines, antibacterial defence, PO activity) and dependence of immune activity on environmental variation (cytokines, antibacterial defence, ROS production), as well as how these responses may depend on general stress reactions (HSP). Interestingly, many examined immune factors (PRRs, lectins, TLR pathway, cytokines, ROS production, apoptosis, PO enzymes, antibacterial proteins) showed considerable among-individual variation in the genetically diverse experimental snails used in this study. In addition to the components responding to immune elicitors, these factors can be important determinants of fitness variation within and between natural snail populations and should be included in future ecoimmunological studies.

## Methods

### Study animals and experimental design

This study employed adult *L. stagnalis* snails (*N* = 48, shell length 24.6–30.5 mm) from an F_2_ generation of a laboratory stock population that was generated by interbreeding snails that originated from seven different natural populations in Switzerland (see [54]). Interbreeding was done to increase genetic variation among the experimental snails relative to natural populations that may show low genetic diversity [90]. Higher genetic diversity among experimental snails increased the potential to identify immune genes that show variation in their transcription among individuals depending, for example, on their genotype and allelic variation.

Before the experiment, experimental snails were placed individually in perforated 0.2 L plastic cups and distributed in four water baths each containing 40 L of aged tap water at 20 °C, with twelve snails per water bath. Snails were fed with fresh lettuce ad libitum*,* and half of the water in each water bath was changed every second day. This set-up allowed water changes with minimal disturbance to the snails. Snails were acclimated to these conditions for 4 (treatments including an environmental change) to 6 days (treatments including an immune challenge) before exposure to experimental manipulations (see the next two paragraphs for the description of the treatments). The acclimation period differed between treatment types to allow simultaneous sampling of snail tissues for RNA extractions (i.e., duration of the treatments was not the same).

In the experiment, the snails were randomly assigned to nine experimental treatments (Table 1). In seven treatments, snails’ responses to immune elicitors, including the effects of handling that were necessary for immune challenge treatments, were tested. Snails in treatment one (untreated controls; *N* = 5) were maintained as during the acclimation period. Snails in treatment two (anaesthetised controls, *N* = 5) were anaesthetised by placing them into 2% diethyl ether for two and a half minutes. These snails were used to examine the effects of snail handling when compared to untreated controls (treatment 1 above) because anaesthesia was needed in following immune challenge treatments. In treatment three (wounding; *N* = 6), each anaesthetised snail was injected with 170 μL of snail saline (see [91]) in the head-foot using a syringe and needle (0.45 mm × 16 mm BD Microlane™ 3, New Jersey, USA). This treatment examined the effect of wounding in activating immune defence when compared to anaesthesia without exposure to immune elicitors (treatment 2 above). The sham injection also served as a control for the treatments involving bacterial injections (described in the next paragraph).

**Table 1:** Summary of the experimental design

Treatment	Treatment type	***N***	Reference/control treatment
Untreated	–	5	–
Anaesthesia	Handling	5	Untreated
Wounding	Handling/immune challenge	6	Anaesthesia
Injection with *E. coli*	Immune challenge	6	Wounding
Injection with *M. lysodeikticus*	Immune challenge	6	Wounding
Injection with healthy snail tissue	Immune challenge	6	Wounding
Injection with trematode-infected snail tissue	Immune challenge	6	Injection with healthy snail tissue
Elevated temperature	Environmental change	4	Untreated
Food deprivation	Environmental change	4	Untreated

Columns present treatments, treatment types, the number of exposed snails, and the specific reference/control treatment used for each treatment group when examining their effects on gene expression

In treatments four and five (bacterial injections; *N* = 6 for each), anaesthetised snails were injected as above with 0.5 mg/mL lyophilised *E. coli* (Sigma-Aldrich, Steinheim, Germany; product number EC11303) or *M. lysodeikticus* (synonym *Micrococcus luteus*; Sigma-Aldrich, Steinheim, Germany; product number M3770) cells in snail saline, respectively. Both Gram-negative (*E. coli*) and Gram-positive (*M. lysodeikticus*) bacteria were used because lipopolysaccharides of Gram-negative bacteria and peptidoglycans of Gram-positive bacteria are recognised by different receptors that activate pathogen-specific immune responses [14, 21, 92–94].

In treatments six and seven (snail/trematode-tissue injections; *N* = 6 for each), anaesthetised snails were injected as above with soluble extracts from healthy or trematode-infected gonads (ovotestes) dissected from *L. stagnalis* snails collected from a pond in Winterthur, Switzerland (47° 28′N, 8° 43′E). In donor snails, parasite infections were evident from the shedding of strigeid and echinostome-type cercariae. Both healthy and infected gonads were disrupted in 1.5 mL Eppendorf tubes using plastic pestles, adding 1 mL of snail saline per 100 mg of tissue. Samples were vortexed and pelleted briefly (2000×g for 5 s). The supernatant was collected from three uninfected and three infected snails and the samples from each snail type were pooled for injections. The treatment using the extract from healthy gonads served as a control for injection with tissue extract from the trematode-infected snail. After injections, all experimental snails were placed back in their original cups with lettuce for 6 hours to allow their immune systems to respond to immune elicitors.

In addition to immune challenges, snails’ responses to environmental changes were tested in two treatments. Snails in treatment eight (elevated temperature; *N* = 4) were placed in water baths where the water temperature was raised to 25 °C during several hours and maintained at an elevated level for 2 days (see [55, 56]). After the transfer, additional snails were added to all water baths to keep the experimental snails continuously at a density of twelve snails per water bath (data were not collected from these extra snails). Snails in treatment nine (food deprivation; *N* = 4) were maintained without food for 2 days. Untreated snails (treatment 1 above) served as controls for these treatments. All snails survived the experimental treatments and displayed normal motility and activities during the experiment.

### RNA extraction, library preparation and sequencing

At the end of the experimental treatments, each snail was removed from its shell, and whole-body soft tissues were submerged in self-made RNAlater solution (see protocol at https://www.protocols.io/view/RNAlater-Recipe-c56y9d) in which they were cut into small pieces. The cut tissues were stored in 10 mL of RNAlater at − 20 °C. Within the next 15 days, all samples were disrupted under liquid nitrogen using mortar and pestle. Total RNA was extracted from each homogenised tissue sample (71–102 mg per snail) using TRIzol reagent (Invitrogen, Carlsbad, CA, USA) according to the manufacturer’s instructions except for conducting RNA wash with 75% EtOH three times. Residual genomic DNA was removed from samples using Turbo DNA-free kit (Ambion, Austin, TX, USA) according to the manufacturer’s instructions. RNA quantity was measured using Qubit 2.0 Fluorometer (Invitrogen, Carlsbad, CA, USA), and sample quality and purity were verified using 2100 Bioanalyzer (Agilent Technologies, Palo Alto, CA, USA; RNA Nano chips) and NanoDrop ND-1000 spectrophotometer (NanoDrop Technologies Inc., Wilmington, DE, USA), respectively. Complementary DNA (cDNA) libraries were constructed for each snail individual using TruSeq Stranded mRNA Sample Preparation Kit (Illumina, San Diego, CA, USA) according to the manufacturer’s instructions. All libraries were sequenced on Illumina NextSeq 500 platform using paired-end reads with 81 nt read length at the Genomics Facility Basel.

### RNA-seq data handling and transcriptome assembly

Raw Illumina reads from all libraries were subjected to adaptor trimming and quality filtering. Reads with Illumina TruSeq adaptors were trimmed using Cutadapt v1.5 (min. Overlap 30 nt and max. Allowed error rate 0.05). Reads were then quality filtered using PRINSEQ-lite v0.20.4 [min. Read length: 50 nt, GC range: 10–90%, mean Q ≥ 5, trim base left and right with Q < 10, no ambiguous nucleotides allowed, trim poly A/T tail (min. 5) from both sequences, and remove duplicate reads]. Finally, Phix sequences with GenBank genome reference NC_001422.1, as well as SSU and LSU rRNA sequences with SILVA (Release 111) reference, were removed using BBDuk v2015.08.21. To generate a *L. stagnalis* de novo reference transcriptome, the cleaned reads from nine libraries (one randomly chosen library per experimental treatment) were combined and assembled with trinity v2.0.6 (parameters: min contig length: 100, min glue: 4, min kmer cov: 4, group pairs distance: 300, path reinforcement distance: 85, normalise reads, normalise max read cov: 50, and normalise by read set). Likely contaminant transcripts from other organisms were removed by mapping all transcripts on the *L. stagnalis* reference genome (GenBank accession number GCA_900036025.1) and removing those that lacked a significant similarity hit in the genome if they were not previously characterised from *L. stagnalis*. Completeness of the produced reference transcriptome was assessed by examining the detection of core BUSCO genes [95, 96] from metazoans (978 genes).

### Annotation of immune-, stress- and metabolism-related factors

Amino acid sequences of previously characterised proteins/genes relevant for immune function, stress responses and metabolism primarily from molluscs (especially gastropods; e.g., [38, 97]), but also from other organisms, were collected from GenBank (Additional file 13) and used in BLAST (v2.6.0+) similarity searches to identify orthologs in the *L. stagnalis* reference transcriptome (226,116 contigs). Components of the immune system queried included non-self recognition through pathogen-recognition receptors (PRRs; all abbreviations are explained at the end of the article), Toll-like receptor (TLR) pathway, cytokines, antibacterial peptides and proteins, production of reactive oxygen species (ROS), phenoloxidase (PO)/melanisation-type defence, and apoptosis. Components of stress responses included cell protection and survival, oxidative stress and antioxidant enzymes. Targeted components linked to metabolism included regulatory proteins, proteins related to the transport of elements and compounds as well as a selection of receptors.

From the statistically best Blast hits (E-value ≤0.05), transcripts that were at least 60% of the length of the shortest reference sequence and showed minimum 40% similarity were used to identify ORFs (EMBOSS v6.6.0.0) that were between a start and a stop codon. ORFs that were at least 60% of the length of the shortest reference sequence (absolute minimum length: 30 amino acids) were examined for the presence of a signal peptide (SignalP v4.1) and the domain structure (SMART, PFAM, NCBI). For identification of fibrinogen-related proteins (FREPs), sequences that were computationally identified to contain fibrinogen-like (FBG) domains were manually inspected for upstream immunoglobulin superfamily (IgSF) domains because the diffuse sequence motifs of invertebrate IgSF domains frequently challenge automated detection.

### Transcript expression level analysis

To examine variation in the expression levels of different transcripts among experimental snails, sequencing reads from each library were first indexed to transcripts in the reference transcriptome using Kallisto v.0.44.0 with 100 bootstraps [98]. Sleuth was then used for downstream processing [99]. The transcriptome-wide expression profiles in different libraries were visualised using their PC scores obtained from a principal component analysis (PCA, the first 5 PCs were used) using the internal normalisation in Sleuth. Very high variation in the expression profiles among snail individuals even within experimental treatments (see the Results section) violated the assumptions of formal statistical tests for differential expression of individual transcripts among treatments, thus negating their use (see [100]). Additionally, we are not aware of a statistical test that compares the amount of variation in transcription over biological replicates across transcripts within treatments. Therefore, both the within- and among-treatment variation in expression levels of individual transcripts that represented the annotated factors were examined visually using heatmaps that presented all experimental snails. Signal strength was calculated in units of transcripts per million (TPM). This measure calculates the proportion of counts per base for each transcript in the whole data set (multiplied by million).

## Supplementary Information

**Additional file 1.** Summary of the annotated transcripts linked to proteins with a role in immune defence, stress-responses, or metabolism. The total number of identified transcripts, the number of transcripts with unique open reading frames (ORFs), an example domain architecture using SMART and PFAM domains (when available in the annotation database), and transcripts representing unique ORFs for each examined factor are presented. Transcript ID the domain architecture refers to is in bold.

**Additional file 2.** Principal component analysis (PCA) plots showing the variation in transcriptome-wide expression profiles of the experimental snails using the first five principal components (PCs) after internal normalisation in Sleuth. Additionally, the proportion of total variance each PC explained in the data is presented.

**Additional file 3.** Expression levels of individual transcripts found to represent annotated factors related to TLR signalling pathway in units of transcripts per million (TPM) for each experimental snail. Heatmap shows the variation for each factor using the dynamic range. Transcripts related to each factor are clustered according to their similarity.

**Additional file 4.** Expression levels of individual transcripts found to represent the annotated antibacterial defence factors in units of transcripts per million (TPM) for each experimental snail. Heatmap shows the variation for each factor using the dynamic range. Transcripts related to each factor are clustered according to their similarity.

**Additional file 5.** Expression levels of individual transcripts found to represent annotated factors related to phenoloxidase/melanisation-type reaction in units of transcripts per million (TPM) for each experimental snail. Heatmap shows the variation for each factor using its dynamic range. Transcripts related to each factor are clustered according to their similarity.

**Additional file 6.** Expression levels of individual transcripts found to represent annotated factors related to non-self recognition in units of transcripts per million (TPM) for each experimental snail. Heatmap shows the variation for each factor using the dynamic range. Transcripts related to each factor are clustered according to their similarity.

**Additional file 7.** Expression levels of individual transcripts found to represent annotated cytokines in units of transcripts per million (TPM) for each experimental snail. Heatmap shows the variation for each factor using the dynamic range. Transcripts related to each factor are clustered according to their similarity.

**Additional file 8.** Expression levels of individual transcripts found to represent annotated factors related to the production of reactive oxygen species (ROS) in units of transcripts per million (TPM) for each experimental snail. Heatmap shows the variation for each factor using the dynamic range. Transcripts related to each factor are clustered according to their similarity.

**Additional file 9.** Expression levels of individual transcripts found to represent annotated factors related to apoptosis in units of transcripts per million (TPM) for each experimental snail. Heatmap shows the variation for each factor using the dynamic range. Transcripts related to each factor are clustered according to their similarity.

**Additional file 10.** Expression levels of individual transcripts found to represent the annotated stress-response factors in units of transcripts per million (TPM) for each experimental snail. Heatmap shows the variation for each factor using the dynamic range. Transcripts related to each factor are clustered according to their similarity.

**Additional file 11.** Expression levels of individual transcripts found to represent the annotated antioxidant enzymes in units of transcripts per million (TPM) for each experimental snail. Heatmap shows the variation for each factor using the dynamic range. Transcripts related to each factor are clustered according to their similarity.

**Additional file 12.** Expression levels of individual transcripts found to represent annotated factors related to metabolism in units of transcripts per million (TPM) for each experimental snail. Heatmap shows the variation for each factor using the dynamic range. Transcripts related to each factor are clustered according to their similarity.

**Additional file 13 **GenBank accession numbers of the sequences of proteins/genes relevant for immune function, stress responses, antioxidation and metabolism that were used as references in BLAST similarity searches to identify orthologs in the *L. stagnalis* reference transcriptome.

## Data Availability

The raw sequencing reads are deposited in the NCBI Sequence Read Archive (accession number PRJNA664475). The reference transcriptome is available in 10.5281/zenodo.4044169.

## Abbreviations

ADH: Alcohol dehydrogenase
AIF: Apoptosis-inducing factor
AMP: Antimicrobial peptide
CAT: Catalase
DUOX: Dual oxidase
DUOXA: Oxidase maturation factor
ERR: Estrogen-related receptor
FAIM1: Fas apoptotic inhibitory molecule 1
FBG: Fibrinogen-like domain
FREP: Fibrinogen-related protein
GNBP: Gram-negative bacteria binding-protein
GST: Glutathione S-transferase
HSF: Heat shock factor
HSP70: 70 kilodalton heat shock protein
HSP90: 90 kilodalton heat shock protein
HTRA2: Serine protease
IAP: Inhibitor of apoptosis proteins
IgSF: Immunoglobulin superfamily
IKK: Inhibitor of NF-κB kinase
IL-17: Interleukin 17
IκB: Inhibitor of nuclear factor-κB
LAAO: L-amino acid oxidase
LBP/BPI: Lipopolysaccharide-binding protein/bactericidal permeability-increasing protein
L-dopa: l-3,4-dihydroxyphenylalanine
LITAF: PS-induced TNF-α factor
LRR: Leucine-rich repeat
MIF: Macrophage migration inhibitory factor
MPEG: Macrophage expressed gene
MyD88: Myeloid differentiation primary response protein 88
NADPH: Nicotinamide adenine dinucleotide phosphate
Nemo: NF- B essential modulator
NF- B: Nuclear factor B
NF-κB: Nuclear factor-κB
NOS: Nitric oxide synthase
NOX: NADPH-oxidase
ORF: Open reading frame
PARP: Poly (ADP-ribose) polymerase
PC: Principal component
PCA: Principal component analysis
PGRP: Peptidoglycan recognition protein
PO: Phenoloxidase
PP1: Protein phosphatase
PRR: Pathogen-recognition receptor
RXR: Retinoid acid receptor
SARM: Sterile alpha and armadillo motif-containing protein
SOD and MnSOD: Superoxidase dismutases
TAB: TAK1 binding protein
TAK1: Transforming growth factor β-activated kinase-1
TIR: Toll-interleukin receptor
TLR: Toll-like receptor
TNF: Tumor necrosis factor
TPM: Transcripts per million
TRAF: Tumor necrosis factor receptor-associated factor
TRF: TRAF-like protein
VIgLs: Variable immunoglobulin and lectin-domain-containing molecules
