# Supplementary material for: Transcriptome profiling of Lymnaea stagnalis (Gastropoda) for ecoimmunological research
Source: BMC Genomics. 2021 Mar 1;22:144. doi: 10.1186/s12864-021-07428-1 (PMC7919325; doi:10.1186/s12864-021-07428-1)
Supplement: Supplementary file 1 — Additional file 1. Summary of the annotated transcripts linked to proteins with a role in immune defence, stress-responses, or metabolism. The total number of identified transcripts, the number of transcripts with unique open reading frames (ORFs), an example domain architecture using SMART and PFAM domains (when available in the annotation database), and transcripts representing unique ORFs for each examined factor are presented. Transcript ID the domain architecture refers to is in bold. [file 12864_2021_7428_MOESM1_ESM.pdf]

Additional file 1 Summary of the annotated transcripts linked to proteins with a role in immune defence, stress-responses, or metabolism. The total number of identified transcripts, the number of transcripts with unique open reading frames (ORFs), an example domain architecture using SMART and PFAM domains[a] (when available in the annotation database), and transcripts representing unique ORFs for each examined factor are presented. Transcript ID the domain architecture refers to is in bold.

Component	# transcripts	# unique ORFs	Example of domain structure	Transcript IDs with unique ORFs
**Non-self recognition**				
PGRPs	8	7	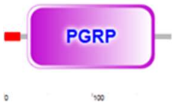	14145_c0_g1_i1<br>**32865_c0_g1_i1**<br>34108_c0_g1_i1<br>36537_c0_g1_i1<br>36963_c0_g1_i1<br>42094_c0_g6_i1<br>42956_c0_g1_i1
GNBPs	8	4	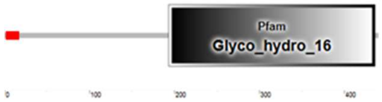	**41774_c0_g1_i2**<br>47227_c0_g2_i1<br>47391_c1_g1_i1<br>48085_c1_g8_i11
FREPs	2	2	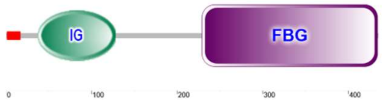	**36764_c0_g1_i2**<br>45250_c0_g1_i1
Galectins	20	6	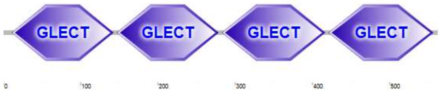	42521_c0_g1_i2<br>44043_c0_g3_i1<br>**44175_c0_g1_i1**<br>47859_c0_g2_i1<br>47859_c0_g2_i5<br>48059_c1_g2_i1
Chi-lectins	11	8	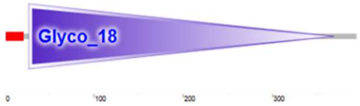	**33218_c0_g1_i1**<br>44081_c0_g1_i1<br>44227_c0_g2_i2<br>45634_c0_g3_i1<br>47306_c0_g5_i1<br>48292_c1_g1_i3<br>26259_c0_g1_i1<br>45255_c1_g3_i3
L-type lectins	3	2	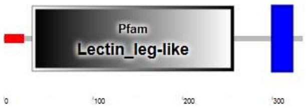	**44959_c0_g1_i1**<br>48268_c2_g1_i1
M-type lectins	9	3	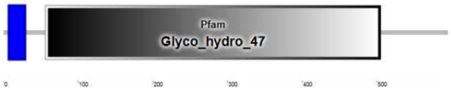	37655_c0_g1_i1<br>39342_c0_g1_i1<br>**47242_c4_g2_i1**
**TLR pathway**				
TLR	1	1	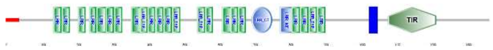	28651_c0_g1_i1

MyD88	7	1	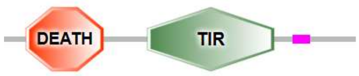	47065_c0_g1_i1
TRAF	1	1	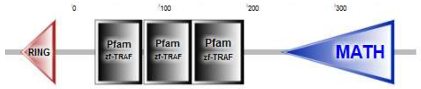	22931_c0_g1_i1
TRF	1	1	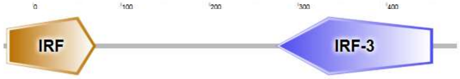	34891_c0_g1_i1
TAB	1	1	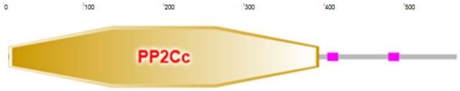	24669_c0_g1_i1
SARM	1	1	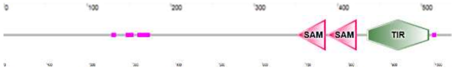	46010_c7_g2_i1
IKKα	2	1	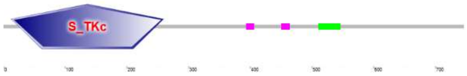	35196_c0_g1_i1
IKKγ Nemo	6	1	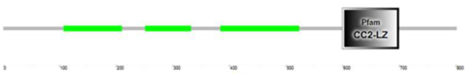	47446_c1_g1_i1
LITAF	6	2	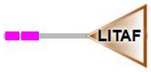	**45095_c0_g1_i1**<br>46794_c1_g1_i1
IκB	2	2	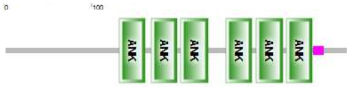	**39554_c0_g1_i1**<br>46270_c2_g2_i1
NF-κBp65	4	2	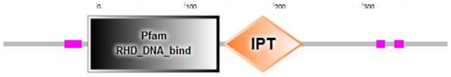	38589_c1_g1_i1<br>**43263_c0_g2_i1**
MPEG	3	2	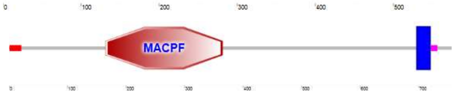	**46200_c0_g1_i1**<br>47480_c0_g1_i1
**Cytokines**				
MIF	2	1	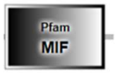	36453_c0_g1_i1
IL-17	3	3	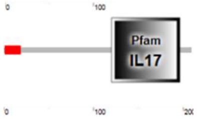	**110902_c0_g1_i1**<br>4951_c0_g1_i1<br>17215_c1_g1_i1
TNF	63	23	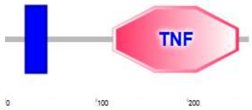	15848_c0_g1_i1<br>**20877_c0_g2_i1**<br>34263_c0_g1_i2<br>37129_c0_g1_i1<br>37407_c0_g1_i1<br>37593_c0_g1_i1<br>39166_c0_g1_i1<br>41927_c0_g1_i1<br>42168_c1_g1_i2<br>42302_c0_g4_i1<br>42612_c1_g1_i1<br>42736_c0_g1_i1<br>43123_c0_g2_i2

				43870_c0_g1_i2<br>44512_c0_g1_i2<br>45558_c0_g3_i1<br>45927_c0_g1_i1<br>46059_c2_g1_i2<br>46501_c1_g2_i1<br>47286_c3_g3_i2<br>47468_c1_g1_i1<br>47690_c7_g2_i10<br>48316_c0_g1_i1
**Antimicrobial defence**				
Macins	7	6	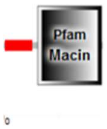	**18198_c0_g1_i1**<br>18890_c0_g1_i1<br>34989_c0_g2_i1<br>35624_c0_g1_i1<br>41887_c0_g3_i1<br>43018_c0_g6_i3
LBP/BPI	4	3	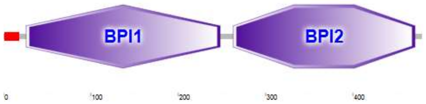	**25950_c0_g1_i1**<br>45368_c0_g6_i1<br>46649_c0_g1_i1
LAAO	6	3	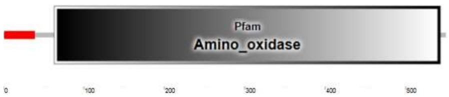	**47821_c4_g3_i1**<br>47893_c0_g9_i1<br>48351_c1_g3_i1
Lysozymes	11	4		28373_c0_g1_i1<br>35313_c1_g1_i1<br>36143_c0_g3_i1<br>44233_c1_g1_i1
Lymnaea-lysins	4	3	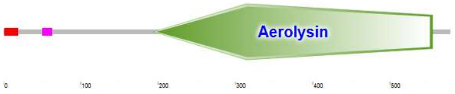	**38389_c0_g1_i1**<br>47349_c1_g38_i2<br>5994_c0_g2_i1
Stagnalysins	3	3		34104_c0_g1_i1<br>42332_c1_g1_i1<br>48069_c5_g1_i1
**Production of reactive oxygen species**				
NOX	9	3	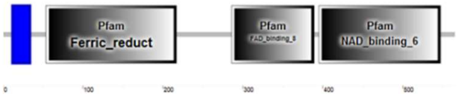	**40220_c1_g2_i1**<br>36496_c0_g1_i1<br>36278_c0_g1_i1
DUOX	6	2	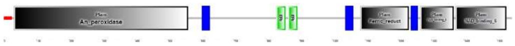	**42865_c0_g1_i3**<br>46890_c0_g1_i1
DUOXA	1	1	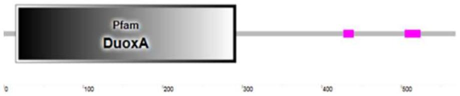	38205_c0_g1_i1
NOS	3	1	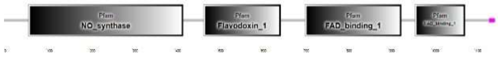	45570_c1_g1_i1
Peroxidasin	16	6		40499_c0_g1_i1<br>45939_c0_g1_i1

				46236_c0_g1_i2<br>46303_c1_g1_i1<br>47256_c0_g1_i1<br>48181_c2_g1_i1
Glutaredoxin	7	4	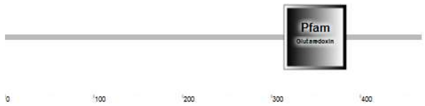	**34293_c0_g2_i1**<br>34343_c1_g2_i1<br>4572_c0_g2_i1<br>45783_c0_g1_i1
GST	32	17	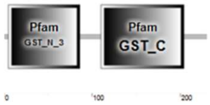	**1429_c0_g2_i1**<br>31273_c0_g1_i1<br>36750_c0_g1<br>38055_c0_g1_i2<br>39155_c0_g1_i1<br>40581_c0_g1_i1<br>40585_c0_g1_i1<br>41000_c0_g2_i1<br>43131_c0_g1_i1<br>44207_c5_g1_i1<br>44723_c0_g5_i1<br>46105_c0_g60_i1<br>46670_c0_g1_i1<br>46742_c1_g1_i1<br>46851_c0_g1_i2<br>46851_c0_g1_i6<br>46929_c1_g35_i1
Nucleoredoxin	7	4	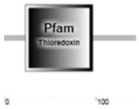	**33046_c0_g1_i1**<br>39371_c0_g1_i1<br>44859_c0_g2_i1<br>45653_c0_g1_i2
Peroxiredoxin	9	5	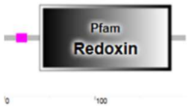	**27522_c0_g1_i1**<br>34959_c0_g2_i1<br>41634_c2_g1_i1<br>46977_c0_g1_i1<br>47425_c0_g2_i6
**Phenoloxidase/melanisation**				
Laccase	19	8	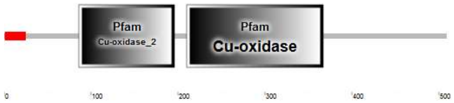	**35966_c0_g1_i1**<br>38462_c0_g1_i1<br>45077_c0_g1_i1<br>47321_c2_g2_i1<br>47844_c2_g5_i1<br>48217_c1_g1_i10<br>38987_c2_g2_i1<br>45762_c4_g1_i3
Tyrosinase	2	2	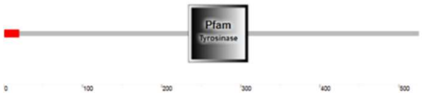	**26246_c2_g1_i1**<br>46270_c3_g2_i1

**Apoptosis**				
FAIM1	1	1	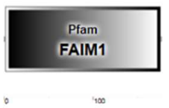	44771_c0_g1_i1
HTRA2	3	2	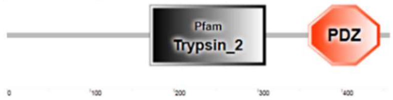	**36021_c0_g1_i1**<br>37936_c0_g1_i1
AIF	1	1	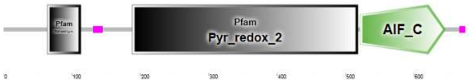	40505_c0_g1_i1
IAP	25	10	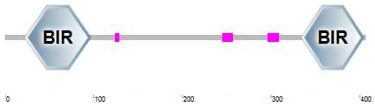	**30243_c0_g1_i1**<br>41110_c0_g1_i1<br>42472_c1_g1_i1<br>46127_c2_g1_i1<br>47200_c0_g1_i1<br>47310_c1_g1_i4<br>47642_c0_g1_i11<br>48019_c3_g3_i1<br>48046_c0_g1_i3<br>48048_c3_g1_i2
Bcl-Bax	1	1	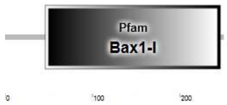	44934_c1_g1_i3
PARP	21	b		
Caspase	20	8	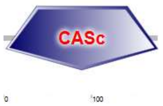	**33457_c0_g1_i1**<br>37005_c0_g1_i1<br>37479_c0_g2_i1<br>42025_c0_g1_i2<br>43539_c0_g1_i1<br>45052_c4_g2_i1<br>48203_c0_g1_i2<br>48216_c2_g3_i10
**Stress responses**				
HSP70	13	5	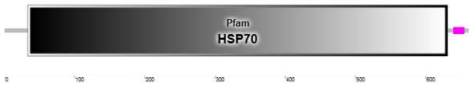	**40510_c1_g2_i1**<br>41463_c0_g1_i1<br>44096_c1_g1_i1<br>46830_c1_g3_i1<br>47977_c0_g5_i1
HSP90	1	1	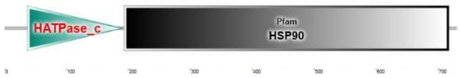	47161_c0_g4_i1
HSF	1	1	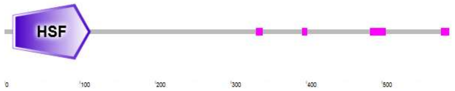	29117_c0_g1_i1
**Antioxidant enzymes**				
SOD	6	2	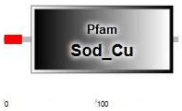	**41920_c0_g1_i2**<br>45899_c0_g2_i1

MnSOD	1	1	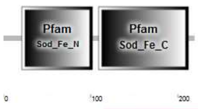	28257_c0_g1_i1
CAT	2	2	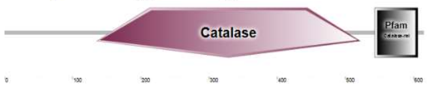	**40830_c0_g1_i1**<br>44469_c0_g1_i1
glutathione peroxidase	5	3	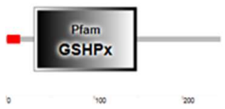	**36171_c0_g2_i1**<br>37235_c0_g1_i1<br>47906_c1_g7_i1
glutathione reductase	1	1	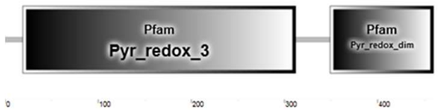	44983_c1_g1_i1
**Metabolism**				
PP1	2	1	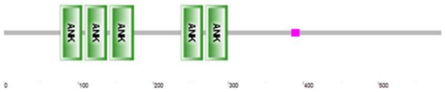	36580_c0_g1_i1
Ubiquitin	13	8	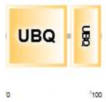	**109816_c1_g1_i1**<br>33535_c0_g1_i1<br>40537_c0_g1_i1<br>44027_c1_g1_i2<br>44915_c1_g1_i1<br>45261_c0_g1_i1<br>46234_c4_g1_i1<br>46544_c1_g3_i2
Ferritin	3	3	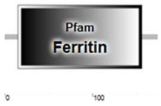	**125081_c0_g1_i1**<br>33209_c0_g1_i1<br>43780_c0_g5_i1
ADH	3	1	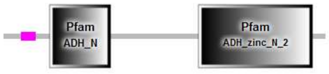	47996_c0_g14_i4
ERR	2	2	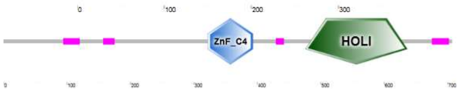	**36130_c1_g1_i4**<br>42943_c0_g1_i1
RXR	5	3	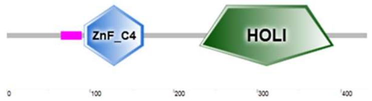	33793_c0_g1_i1<br>41983_c1_g1_i1<br>42786_c1_g1_i1

[a]SMART/PFAM domains: ADH_N – alcohol dehydrogenase GroES-like domain; ADH_zinc_N_2 – zinc-binding dehydrogenase; Aerolysin – aerolysin toxin; AIF_C – apoptosis-inducing factor, mitochondrion-associated, C-term; Amino_oxidase – flavin-containing amine oxidoreductase; An_peroxidase – animal heme-dependent peroxidase; An_peroxidase – animal heme-dependent peroxidase; ANK – ankyrin repeats; Bax1-I – inhibitor of apoptosis-promoting Bax1; BIR – baculoviral inhibition of apoptosis protein repeat; BPI1 – bactericidal permeability-increasing protein / lipopolysaccharide-binding protein / cholesteryl ester transfer protein N-terminal domain; BPI2 – bactericidal permeability-increasing protein / lipopolysaccharide-binding protein / cholesteryl ester transfer protein C-terminal domain; CASc – caspase, interleukin-1 beta converting enzyme homologues; Catalase – catalase for enzymatic detoxification of hydrogen peroxide; CC2-LZ – leucine zipper of domain CC2 of NEMO, NF-kappa-B essential modulator; Cu-oxidase – multicopper oxidase; DEATH – DEATH domain, bind each other to form oligomers of factors

involved in signaling pathways, frequently associated with immunity or apopotosis; Deltameth_res – deltamethrin resistance; DuoxA – dual oxidase maturation factor; EHf – EF-hand, calcium binding motif; FAD_binding_1 – flavoprotein pyridine nucleotide cytochrome reductase; FAD_binding_8 – FAD binding domain associated with ferric reductase NAD binding proteins and the heavy chain of Cytochrome b-245; FAD_binding_8 – FAD-binding domain; FAIM1 – Fas apoptotic inhibitory molecule; FBG – fibrinogen-like domain; Ferric_reduct – ferric reductase like transmembrane component; Ferritin – ferritin-like domain; Flavodoxin_1 – flavodoxin-like domain; GLECT – galectin; Glutaredoxin – glutaredoxin, oxidation repair enzyme; Glyco_18 – O-Glycosyl hydrolases; Glyco_hydro_16 – glycosyl hydrolases family 16; Glyco_hydro_47 – glycoside hydrolase family 47; GSHPx – glutathione peroxidase; GST_C – glutathione S-transferase, C-terminal domain; GST_N_3 – glutathione S-transferase; HATPase_c – histidine kinase-like ATPases; HOLI – ligand binding domain of hormone receptors; HSF – heat shock factor; HSP70 – heat shock protein 70; HSP90 – heat shock protein 90; IG – immunoglobulin; IL17 – interleukin-17; IPT – ig-like, plexins, transcription factors; IRF – interferon-regulatory factor; IRF-3 – interferon-regulatory factor 3; Lectin_leg-like – L-type lectin domain; LITAF – LPS-induced TNF-activating factor; LRR – leucine-rich repeats; Macin – macin family; MACPF – membrane-attack complex / perforin; MATH – meprin and TRAF homology; MIF – macrophage migration inhibitory factor; NAD_binding_1 – oxidoreductase NAD-binding domain; NAD_binding_6 – ferric reductase NAD binding domain; NO_synthase – nitric oxide synthase; PBRP – animal peptidoglycan recognition protein; PDZ – domain present in PSD-95, Dlg, and ZO-1/2, proteins that anchor receptor proteins to the cytoskeleton, to help form signal transduction complexes; PP2Cc – serine/threonine phosphatases family 2C, catalytic domain; Pyr_redox_2 – pyridine nucleotide-disulphide oxidoreductase; Pyr_redox_3 – pyridine nucleotide-disulphide oxidoreductase; Pyr_redox_dim – pyridine nucleotide-disulphide oxidoreductase, dimerisation domain; Redoxin – redoxin domain with oxidoreductase activity; RHD_DNA_bind – Rel homology DNA-binding domain; RING – ring finger; S_TKc – serine/Threonine protein kinases, catalytic domain; SAM – sterile alpha motif; Sod_Cu & Sod_Fe_N & Sod_Fe_C – catergories of superoxide dismutase; Thioredoxin – thioredoxin, small redox proteins involved in responses to reactive oxygen species and cell-cell communication; TIR – Toll/interleukin-1 receptor; TNF – tumour necrosis factor family; Trypsin_2 – trypsin-like peptidase domain; Tyrosinase – tyrosinase, enzymatic domain of copper-type 3 enzymes involved in melanization contributing to pigmentation or immune defence; UBQ – ubiquitin homologues; zf-TRAF – TRAF-type zinc finger; ZnF_C4 – c4 zinc finger in nuclear hormone receptors.

[b]Determining which sequences cover a complete ORF not possible.
