# Supplementary material for: Transcriptome profiling of Lymnaea stagnalis (Gastropoda) for ecoimmunological research
Source: BMC Genomics. 2021 Mar 1;22:144. doi: 10.1186/s12864-021-07428-1 (PMC7919325; doi:10.1186/s12864-021-07428-1)
Supplement: Supplementary file 2 — Additional file 2. Principal component analysis (PCA) plots showing the variation in transcriptome-wide expression profiles of the experimental snails using the first five principal components (PCs) after internal normalisation in Sleuth. Additionally, the proportion of total variance each PC explained in the data is presented. [file 12864_2021_7428_MOESM2_ESM.pdf]

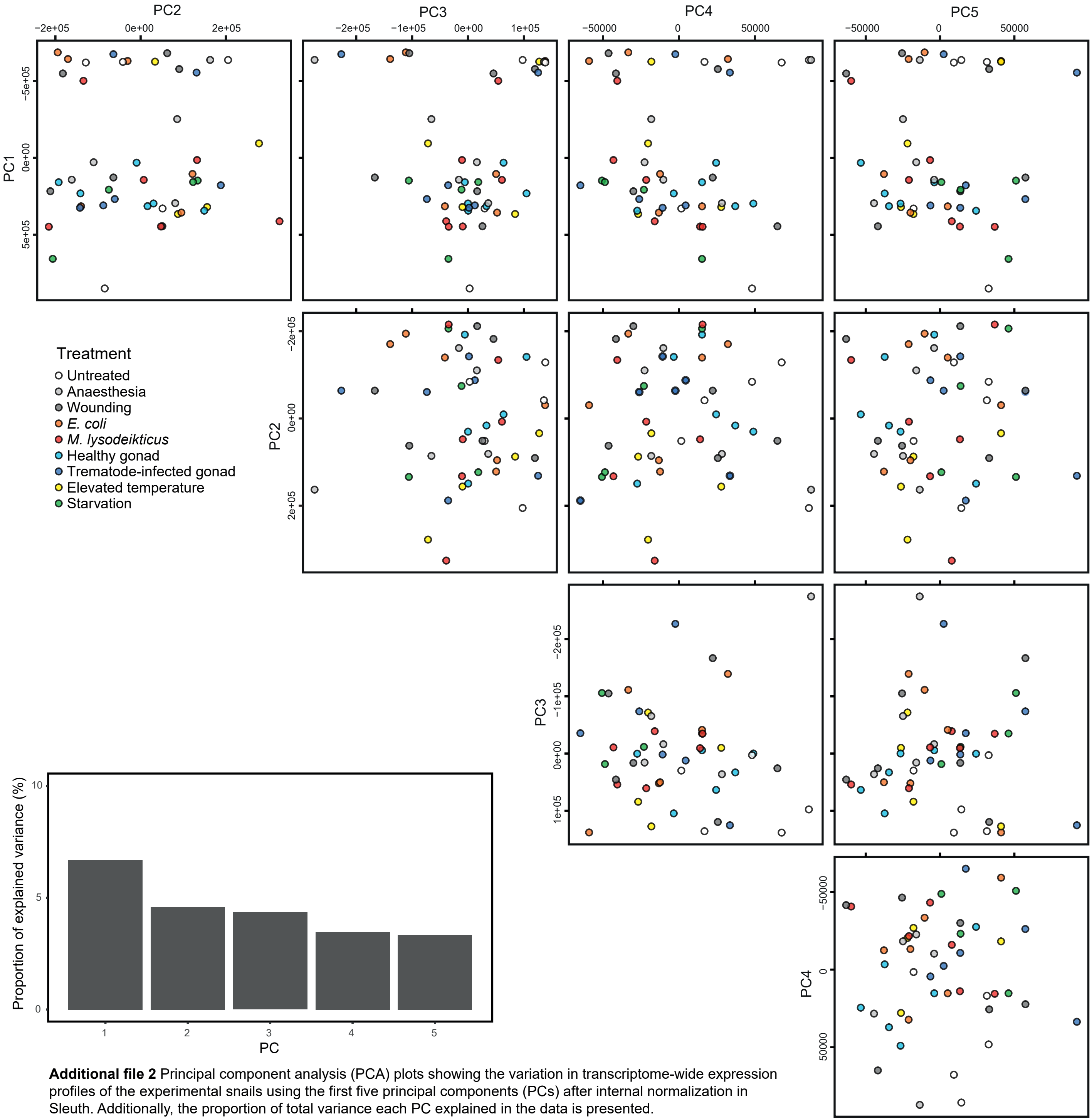

Additional file 2 Principal component analysis (PCA) plots showing the variation in transcriptome-wide expression profiles of the experimental snails using the first five principal components (PCs) after internal normalization in Sleuth. Additionally, the proportion of total variance each PC explained in the data is presented.
