# Supplementary material for: Transcriptome profiling of Lymnaea stagnalis (Gastropoda) for ecoimmunological research
Source: BMC Genomics. 2021 Mar 1;22:144. doi: 10.1186/s12864-021-07428-1 (PMC7919325; doi:10.1186/s12864-021-07428-1)
Supplement: Supplementary file 9 — Additional file 9. Expression levels of individual transcripts found to represent annotated factors related to apoptosis in units of transcripts per million (TPM) for each experimental snail. Heatmap shows the variation for each factor using the dynamic range. Transcripts related to each factor are clustered according to their similarity. [file 12864_2021_7428_MOESM9_ESM.pdf]

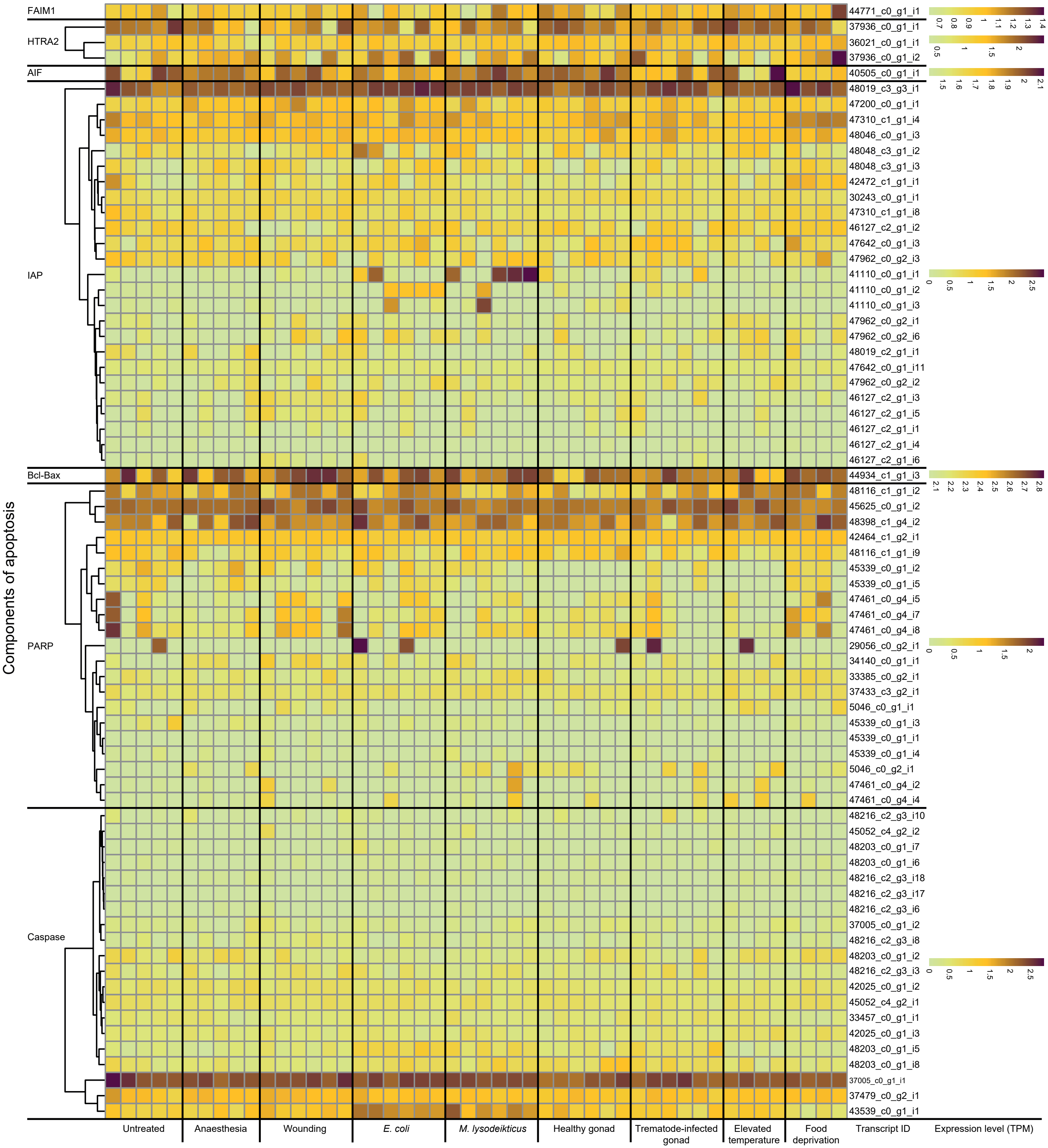

Additional file 9 Expression levels of individual transcripts found to represent annotated factors related to apoptosis in units of transcripts per million (TPM) for each experimental snail. Heatmap shows the variation for each factor using the dynamic range. Transcripts related to each factor are clustered according to their similarity.
