# Supplementary material for: Transcriptome profiling of Lymnaea stagnalis (Gastropoda) for ecoimmunological research
Source: BMC Genomics. 2021 Mar 1;22:144. doi: 10.1186/s12864-021-07428-1 (PMC7919325; doi:10.1186/s12864-021-07428-1)
Supplement: Supplementary file 13 — Additional file 13 GenBank accession numbers of the sequences of proteins/genes relevant for immune function, stress responses, antioxidation and metabolism that were used as references in BLAST similarity searches to identify orthologs in the L. stagnalis reference transcriptome. [file 12864_2021_7428_MOESM13_ESM.pdf]

Additional file 13 GenBank accession numbers of the sequences of proteins/genes relevant for immune function, stress responses, antioxidation and metabolism that were used as references in BLAST similarity searches to identify orthologs in the *L. stagnalis* reference transcriptome.

Component	Reference(s)
**Non-self recognition**	
PGRP	AEH26026.1, AHB30456.1, XP_013064763.1, XP_013064406.1, XP_013062325.1, ABO40832.1, ABK76645.1, NP_001298227.1, XP_013063186.1, XP_013062465.1, XP_013062456.1, XP_005100262.1, XP_005093494.1, ABO40831.1, ABO40830.1, ABO40829.1
GNBP	ABO40828.1
FREP	Q8VCM7.1, ADA82222.1, CBM41040.1, AEO50745.1, O70165.1
Galectin	ACO36044.1, NP_034837.2, ABQ09359.1, ACO53607.1, BAF75419.1
Chi-lectin	O35744.2, P36222.2, Q1RQ16, Q1RQ22
C-type lectins	AAX19697.1, ACS72237.1, AFA36633.1, ABO26594.1, P11226.2
F-box lectins	Q96EF6.1, Q80UW2.1
F-type lectins	Q7SIC1.1, ACF94293.1, ADJ40024.1, AFO64339.2, ADR63290.1
I-type lectins	Q9Y286.1, AAH47244.1, AAA20148.1, P13597.1, Q91Y57.3
L-type lectin	P49257.2, Q12907.1, EKC31192.1, EKC20002.1
M-type lectin	Q925U4.1, Q91VV3, EKC26076.1, EKC23948.1
P-type lectins	CAA45423.1, EKC18244.1, NP_000867.2, EKC35005.1
R-type lectins	BAA36393.1, BAL61208.1, Q2HZ94
Intelectins	ACC62155.1, CAJ32677.2, ACC62157.1, ACC62156.1, NP_001082570.1
Pentraxins	AAZ68032.1, ABG37054.1, P02743.2, P02741.1
**TLR pathway**	
TLR	AGB93809.1
MyD88	XP_013086371.1, XP_013086405.1
IRAK	XP_013088618.1
TRAF	XP_013069680.1, XP_013069680.1
TBK	XP_013083625.1
TAK	XP_013073733.1, XP_013073731.1, XP_013073732.1, XP_013073736.1, XP_013073734.1, XP_013073735.1
TRF	XP_013089600.1, XP_013073636.1, XP_005088853.1, XP_005104410.1, XP_012934632.1
TAB	XP_013096269.1, XP_013096268.1, XP_005094474.1, XP_005094473.1, XP_014776667.1
SARM	XP_013080872.1, XP_013080871.1

IKKα	XP_005096610.2
IKKγ Nemo	XP_013073873.1
LITAF	XP_013089264.1, XP_013089261.1, XP_013089265.1, XP_013089270.1, XP_013089262.1, XP_013089269.1
IκB	NP_001298198.1
NF-κBp65	NP_001298191.1, ACN73461.1
NF-κBp100	XP_022318641.1, XP_022318640.1
MPEG	XP_013065342.1
**Cytokines**	
MIF	NP_001298224.1, XP_013075404.1, XP_005103629.2, XP_012939653.1, AQY19126.1
IL17	XP_013096467.1, XP_013090467.1, XP_013090466.1, XP_013085850.1, XP_013085675.1, XP_013080675.1, XP_013065647.1, XP_021365521.1, AGZ03660.1, XP_021365518.1, XP_009058928.1, XP_021365519.1, XP_022315060.1, XP_022310682.1, AKM49935.1, XP_009048356.1, XP_005097238.1, XP_011438620.1, NP_001292220.1, EKC33705.1, AKM49938.1, XP_022286467.1, XP_021365533.1, XP_022344404.1, XP_022344403.1, NP_001295831.1, OWF44912.1, OWF56800.1, XP_021364761.1, XP_011438445.1, AKQ98198.1, XP_009051888.1, NP_001295781.1, AKQ98197.1, XP_011414373.1, XP_022318598.1, XP_022315342.1
TNF	XP_013076951.1, XP_013061250.1, XP_013061249.1, XP_013061248.1, XP_013061247.1, XP_013096914.1, XP_013096721.1, XP_013096720.1, XP_013096719.1, XP_013096712.1, XP_013096711.1, XP_013096710.1, XP_013094785.1, XP_013094784.1, XP_013094783.1, XP_013094495.1, XP_013094494.1, XP_013086591.1, XP_013082590.1, XP_013082589.1, XP_013082588.1, XP_013082587.1, XP_013076957.1, XP_013073494., XP_013073185.1, XP_013072204.1, XP_013072202.1, XP_013070598.1, XP_013067540.1, XP_013067539.1, XP_013067535.1, XP_013070598.1, XP_013077259.1, XP_022338094.1, XP_022338095.1, XP_011450914.1, EKC35160.1, XP_021346927.1, XP_021364181.1, XP_013073494.1, XP_012936811.1, XP_021346926.1, OWF56727.1, XP_021340535.1
**Antimicrobial defence**	
Macins	AFR36920.1, CK989857.1
LBP/BPI	AGG82435.1, AKM45823.1, AKM45822.1, AKM45821.1, AKM45820.1
LAAO	NP_001191524.1, AAN78211.1, Q17043.1, P35903.1, AAR14185.1, AAR14187.1, AAR14186.1
Lysozymes	AGQ50333.1, AGQ50332.1, AGQ50331.1, ADR70996.1, ADR70995.1, AGQ50337.1, AGQ50335.1, AGQ50330.1, ADV36303.1, AGQ50336.1, AGQ50334.1, AOX15710.1, AOX15709.1, AOX15708.1, AOX15707.1, XM_013226064.1

Biomphalysin	AGG38744.1, NP_001298219.1
Glabralysin	A0A2C9L301, A0A2C9M7U6, A0A2C9M7H5, A0A2C9L4Y1, A0A2C9LNR7, A0A2C9KPA0
**Production of reactive oxygen species**	
NOX	XP_005090645.1, XP_005096802.1, XP_012938230.1
DUOX	XP_012942535.1, XP_012943483.1
DUOXA	XP_021362528.1, XP_022343917.1
NOS	NP_001191470.1, AGI44587.1
Peroxidasin	XP_013088968.1, XP_005110224.1
Glutaredoxin	XP_013084576.1, XP_013061972.1, XP_005104531.1, XP_005110052.1
GST	XP_013075526.1, XP_012944480.1, XP_013071551.1, XP_013060671.1, XP_012945427.1, RUS76363.1, XP_013097111.1, AEI27296.1, XP_005109181.1, XP_013070482.1, AXN72681.1, XP_013095077.1, XP_009049471.1, RUS84494.1, XP_005094586.1, XP_005106935.1, XP_012941915.1
Nucleoredoxin	EKC27452.1, XP_013073587.1, XP_005098394.1, XP_013074893.1
Peroxiredoxin	XP_013086205.1, XP_013087397.1, XP_013091924.1, ACI42883.1
**Phenoloxidase/melanisation-type reaction**	
proPO	CCQ18551.1, NP_476812.1, AGC54940.1, BAF98646.1
Laccase	XP_013079632.1, XP_013079631.1, XP_013086277.1, XP_013074984.1, XP_013074983.1, XP_013074982.1, XP_013068526.1, XP_013085808.1, XP_013062555.1, XP_013064545.1, XP_013079036.1, XP_013085832.1, XP_013085833.1, XP_013085834.1, XP_013088628.1, XP_013090126.1, XP_013067361.1, XP_013067388.1, XP_013074978.1, XP_013074979.1, XP_013074980.1, XP_013074981.1, ESO98873.1, ESO97324.1, ESO96136.1, ESO89911.1, XP_009059385.1, XP_009053251.1, XP_009051926.1, XP_009050501.1, ESO89918.1, XP_009059392.1, ESO90420.1, ESO89919.1, ESO84603.1, XP_009064708.1, XP_009059393.1, XP_009058892.1, XP_012937904.1, XP_012944262.1, XP_012942999.1, XP_012942636.1, XP_012940646.1, XP_012945653.1, XP_012934797.1
Tyrosinase	XP_013080599.1, XP_013065857.1, APC92582.1, EKC35331.1, EKC38463.1, OWF48254.1, APC92581.1, XP_021374137.1, AMB26746.1, ASR73340.1, ALG64484.1
**Apoptosis**	
FAIM1	XP_013071009.1
HTRA2	XP_013072351.1
AIF	XP_013095497.1, XP_013095498.1
IAP	XP_013075317.1, XP_013064429.1, XP_013064681.1, XP_013075313.1, XP_013060630.1, XP_013090049.1, XP_013064690.1, XP_013075320.1, XP_013082134.1, XP_013084114.1, XP_013084111.1

Bcl-Bax	XP_013075909.1, XP_013089117.1
PARP	XP_013090488.1, XP_013062683.1, XP_013062670.1, XP_013065538.1, XP_013068782.1, XP_013062696.1, XP_013067682.1, XP_013092390.1, XP_013096616.1
Caspase	XP_013067788.1, XP_013065656.1, XP_013073999.1, XP_013074008.1, XP_013088924.1, XP_013094549.1, XP_013095352.1
**Stress responses**	
HSP70	XP_013081556.1, XP_013082114.1, ABB45831.1, XP_013091813.1
HSP90	XP_013072750.1
HSF	XP_013085217.1
**Antioxidant enzymes**	
SOD	AAP93637.2, XP_013070344.1
MnSOD	NP_001298192.1
CAT	ACO59957.1
Glutathione peroxidase	ACO59958.1
Glutathione reductase	ACO59956.1
**Metabolism**	
PP1	XP_013085822.1
Ubiquitin	NP_001298238.1
Ferritin	XP_013080834.1
ADH	XP_013073559.1
ERR	XP_013094385.1, XP_013080351.1
RXR	NP_001298239.1
